# Transcutaneous Carbon Dioxide Decreases Immunosuppressive Factors in Squamous Cell Carcinoma In Vivo

**DOI:** 10.1155/2021/5568428

**Published:** 2021-07-02

**Authors:** Nanae Yatagai, Takumi Hasegawa, Rika Amano, Izumi Saito, Satomi Arimoto, Daisuke Takeda, Yasumasa Kakei, Masaya Akashi

**Affiliations:** Department of Oral and Maxillofacial Surgery, Kobe University Graduate School of Medicine, Kobe, Hyogo, Japan

## Abstract

**Introduction:**

In recent years, the tumour immunosuppressive mechanism has attracted attention as a cause of tumour chemoresistance. Although chemoresistance and immunosuppression of tumours have been reported to be associated with a hypoxic environment, effective treatments to improve hypoxia in tumours have not yet been established. We have previously applied carbon dioxide (CO_2_) to squamous cell carcinoma and have shown that improvement in local oxygenation has an antitumour effect. However, the effects of local CO_2_ administration on tumour immunosuppression, chemoresistance, and combination with chemotherapy are unknown. In this study, we investigated the effects of local CO_2_ administration on squamous cell carcinoma and the effects of combined use with chemotherapy, focusing on the effects on tumour immunosuppressive factors.

**Methods:**

Human oral squamous cell carcinoma (HSC-3) was transplanted subcutaneously into the back of a nude mouse, and CO_2_ and cisplatin were administered. After administration twice a week for a total of 4 times, tumours were collected and the expression of tumour immunosuppressive factors (PD-L1, PD-L2, and galectin-9) was evaluated using real-time polymerase chain reaction and immunostaining.

**Results:**

Compared with the control group, a significant decrease in the mRNA expression of PD-L1 was observed in both, CO_2_-treated and combination groups. Similarly, the expression of PD-L2 and galectin-9 decreased in the CO_2_-treated and combination groups. Furthermore, immunostaining also showed a significant decrease in the protein expression of tumour immunosuppressive factors in the CO_2_-treated and combination groups.

**Conclusion:**

It was confirmed that the tumour immunosuppressive factors decreased due to local CO_2_ administration to the mouse model. CO_2_ administration has the potential to improve the hypoxic environment in tumours, and combined use with chemotherapy may also improve tumour immunosuppression.

## 1. Introduction

The currently available main standard treatments for head and neck cancers are surgery, radiotherapy, and chemotherapy. Among them, chemotherapy is often selected in combination with radiotherapy as either radical or additional postoperative treatment. In addition, chemotherapy is often used to treat recurrent and metastatic cancer. However, resistance to chemotherapy is an important problem in cancer treatment.

Hypoxia is one of the most important factors that cause chemotherapy resistance. For head and neck squamous cell carcinoma (HNSCC), hypoxia-induced drug resistance has been reported with cisplatin administration [1, 2]. One of the important factors involved in hypoxia-induced drug resistance and especially chemotherapy resistance is hypoxia-inducible factor-1 (HIF-1), which acts through the development of hypoxia. HIF-1 transcriptional targets may induce drug resistance by affecting drug transporters [3–7].

In recent years, tumour immunosuppression, in which cancer avoids the immune system of the living body, has received attention as a cause of cancer treatment resistance. Tumour immunosuppression is also related to hypoxia [8–10]. A hypoxic environment and increasing levels of HIF-1 affect the expression of immunosuppressive factors such as programmed death-ligand 1 (PD-L1) and programmed death-ligand 2 (PD-L2) in the tumour microenvironment [11, 12]. Shen et al. reported that PD-L1 may be strongly associated with the development of cisplatin resistance in HNSCC cell lines [13]. Therefore, improvement of the hypoxic environment in cancer tissues is important for cancer treatment, and various approaches have been attempted to achieve this [8]. However, effective treatments for improving tumour hypoxia have not yet been established. Methods need to be developed for improving the hypoxic environment in the tumour effectively and efficiently.

Carbon dioxide (CO_2_) therapy is generally known for improving the hypoxic environment. This effect of CO_2_ is mainly caused by an increase in blood flow and a partial increase of O_2_ pressure in the local tissue; this is known as the Bohr effect [14]. In previous studies, we developed a CO_2_ administration method, which can allow efficient local absorption of CO_2_ [14, 15]. In this method, the CO_2_-absorbing hydrogel allows absorption of CO_2_ gas through the skin, and the pH of the solution decreases depending on the volume of absorbed CO_2_ (H_2_O + CO_2_⟶H^+^ + HCO_3_^−^). A study using near-infrared spectroscopy demonstrated that this transcutaneous application of CO_2_ upregulates O_2_ pressure in the local tissue [14]. We also applied CO_2_ to SCC *in vivo* and discovered that it improved local oxygenation in the tumour [16, 17]. However, the effects of local CO_2_ administration on tumour immunosuppression and chemoresistance, when used alone and in combination with chemotherapy, are unknown. We hypothesised that improving the hypoxic environment by CO_2_ administration would decrease immunosuppressive factors such as PD-L1, PD-L2, and galectin-9 and improve chemoresistance.

In this study, we aimed to investigate the effects of local CO_2_ administration on squamous cell carcinoma and the effects of its combined use with chemotherapy.

## 2. Materials and Methods

### 2.1. Cell Culture

The oral cancer cell line HSC-3 was obtained from the Health Science Research Resources Bank (Osaka, Japan). It was established from a metastatic deposit of poorly differentiated SCC of the tongue in a midinternal jugular lymph node from a 64-year-old man [18]. HSC-3 cells were cultured in Eagle's minimum essential medium (Sigma-Aldrich, St. Louis, MO, USA) supplemented with 10% foetal bovine serum (Sigma-Aldrich) and 1000 units/mL penicillin/streptomycin solution (Sigma-Aldrich). Trypsin (0.25%) and ethylenediaminetetraacetic acid (0.02%; Sigma-Aldrich) solutions were used to isolate cells for subculture, as previously described [16, 19].

### 2.2. Animal Models

Male athymic BALB/cAJcl-nu/nu nude mice aged 7 weeks were obtained from CLEA Japan (Tokyo, Japan). The animal experiments were approved by the Institutional Animal Care and Use Committee (Permission number: P-170402) and were performed in accordance with the Guidelines for Animal Experimentation at Kobe University Animal Experimentation Regulations. HSC-3 cells (4 × 10^6^ cells in 300 mL Eagle's minimum essential medium) were injected subcutaneously into the dorsal region of the mice.

### 2.3. Transcutaneous CO_2_ Treatment

**Figure 1:**
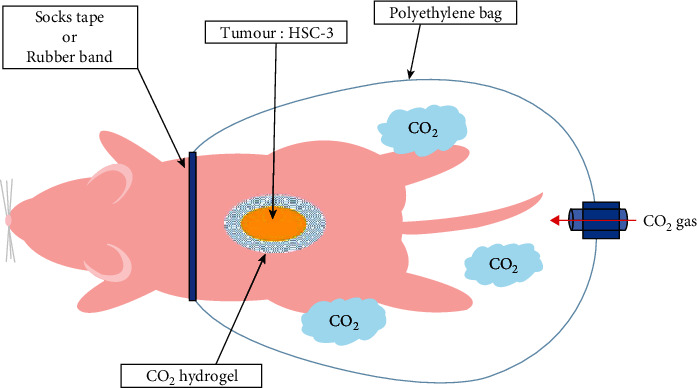
Transcutaneous CO_2_ treatment. The skin around the implanted tumour was covered with CO_2_ hydrogel and sealed with a polyethylene bag, through which 100% CO_2_ gas was administered. Treatment commenced 14 days after HSC-3 cell implantation and was performed twice a week for 2 weeks.

As previously described, the area of skin around the implanted tumour was covered with a CO_2_absorption-enhancing hydrogel (CO_2_ hydrogel), and this area was then sealed with a polyethylene bag; 100% CO_2_ gas was then pumped into the bag [16] (Figure 1). Transcutaneous CO_2_ treatment was applied for 20 min, following which the hydrogel was gently wiped off the skin. Control animals were treated similarly, with room air replacing the CO_2_ [16, 20–22].

### 2.4. Cisplatin (CDDP) Treatment

Cisplatin (Randa Inj. Nippon Kayaku, Tokyo, Japan) was injected intraperitoneally at a dose of 4 mg/kg twice a week for 2 weeks [23, 24].

### 2.5. In Vivo HSC-3 Tumour Studies

Forty mice were randomly divided into four groups: a control group (*n* = 10), a CO_2_-treated group (*n* = 10), a CDDP-treated group (*n* = 10), and a CO_2_ and CDDP combination-treated (combination) group (*n* = 10). Treatment commenced 14 days after HSC-3 cell implantation and was performed twice a week for 2 weeks. Tumour volume and body weight were monitored twice weekly until the end of treatment; tumour volume was calculated according to the formula *V* = *π*/6 × *a*^2^ × *b*, where *a* and *b* represent the shorter and longer diameters of the tumour, as previously described [16, 20–22]. At 24 hours after the end of treatment, the mice were weighed and sacrificed, and the tumours were removed. Immediately after dissection, single-cell suspensions were processed from half the tumour, and RNA was extracted. The other half of the tumour was formalin-fixed and paraffin-embedded for staining. Serial 10 mm thick transverse sections were prepared from each block [16].

### 2.6. Quantitative Real-Time Polymerase Chain Reaction

Primers for *β*-actin, which is the housekeeping gene, were designed as follows: forward (5′-GAT GAG ATT GGC ATG GCT TT-3′) and reverse (5′-CAC CTT CAC CGT TCC AGT TT-3′), which was purchased from Invitrogen (Carlsbad, CA, USA) [16]. Primers for PD-L1, PD-L2, and galectin-9 were purchased from Sino Biological Inc. (Beijing, China). mRNA expression of *β*-actin, PD-L1, PD-L2, and galectin-9 was analysed using quantitative real-time polymerase chain reaction (PCR). Total RNA was extracted from the samples using 500 *μ*L of the TRIzol reagent (Invitrogen) per 10 mg of thinly sliced tissue and cleaned using an RNeasy Mini Kit (Qiagen, Valencia, CA, USA). cDNA was synthesised (1000 ng of total RNA) using a High-Capacity cDNA Reverse Transcription Kit (Applied Biosystems, Foster City, CA, USA). mRNA expression was analysed by quantitative real-time PCR. Quantification of mRNA transcription was performed using an Applied Biosystems StepOne Real-Time PCR System (Applied Biosystems). Reaction conditions included 95°C for 10 min, followed by 40 cycles at 95°C for 15 s and at 60°C for 1 min. The level of each target gene was normalised to the *β*-actin level and expressed relative to the levels of the control group (*ΔΔ*CT methods; Applied Biosystems) [16].

### 2.7. Immunohistochemical Staining

For immunohistochemical staining, formalin-fixed and paraffin-embedded tumour sections were pretreated with pH 9 Tris/EDTA buffer for 40 min at 95°C, quenched with 0.05% H_2_O_2_, and incubated overnight at 4°C with the following primary antibodies in Can Get Signal Immunostain Solution A (Toyobo, Osaka, Japan): PD-L1 polyclonal antibody (Invitrogen), PD-L2 polyclonal antibody (Invitrogen), and galectin-9 polyclonal antibody (Invitrogen). Following this, sections were incubated with horseradish peroxidase- (HRP-) conjugated goat anti-rabbit IgG polyclonal antibody (Nichirei Bioscience, Tokyo, Japan) for 30 min at room temperature. Signals were developed as a brown reaction product using peroxidase substrate 3,3′-diaminobenzidine (Nichirei Bioscience). The sections were counterstained with haematoxylin and examined under a BZ-8000 confocal microscope (Keyence, Osaka, Japan). Immunohistochemical staining was quantified using Hybrid cell count BZ-H3C software (Keyence) [16].

### 2.8. Statistical Analysis

Data are presented as the mean ± standard error. The results were analysed using Kruskal-Wallis and Steel-Dwass tests; the level of statistical significance was set at *p* < 0.05.

## 3. Results

### 3.1. Body Weight

**Figure 2:**
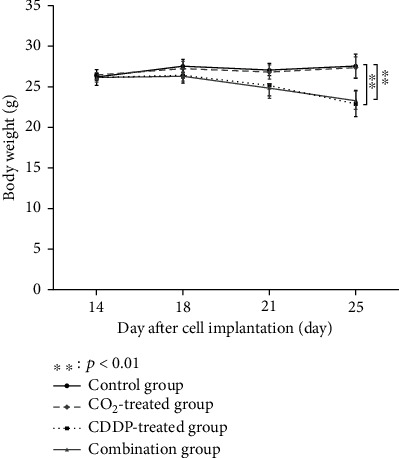
The average body weight of the mice at each time point. Body weight was monitored twice a week for 2 weeks from the start of treatment.

In the CDDP-treated and combination groups, the body weight of the mice at the end of the intervention was significantly reduced compared to that in the control group. In contrast, no significant change in body weight was observed in the CO_2_-treated group after the intervention (Figure 2).

### 3.2. Tumour Size

**Figure 3:**
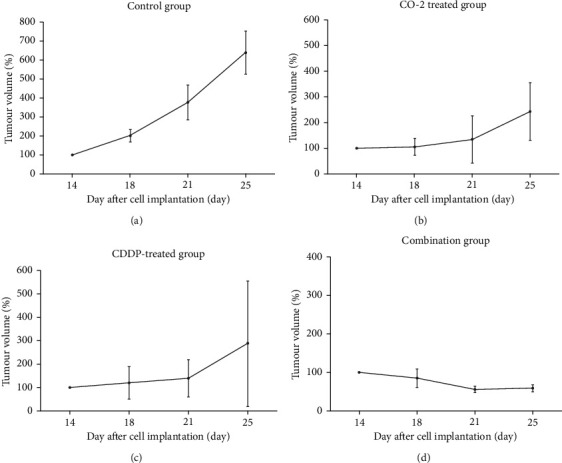
Tumour growth rate in the control group (a), CO_2_-treated group (b), CDDP-treated group (c), and combination group (d). Tumour size was monitored twice a week for 2 weeks from the start of treatment, and tumour volume was calculated according to the formula *V* = *π*/6 × *a*^2^ × *b*, where *a* and *b* represent the shorter and longer diameters of the tumour. The tumour growth rate was calculated based on the tumour volume 14 days after cell transplantation.

After 14 days, we found a significant decrease in tumour volume in the CO_2_-treated, CDDP-treated, and combination groups, compared to the control group. The combination group demonstrated the smallest increase in tumour volume (Figure 3).

### 3.3. Gene Expression

**Figure 4:**
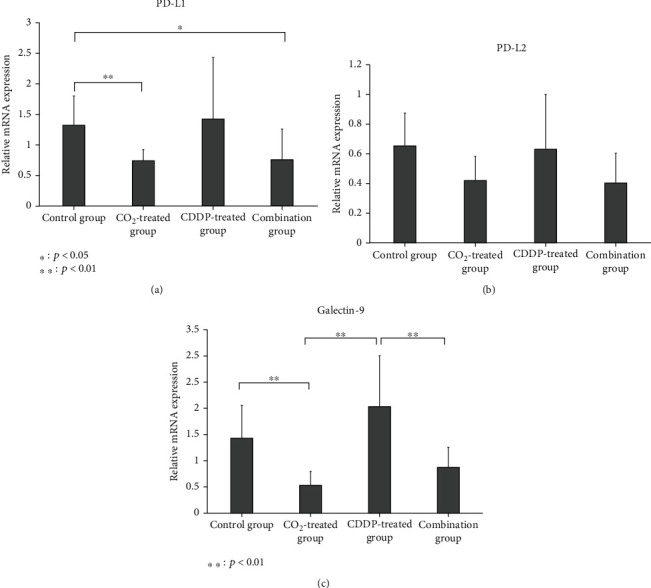
Relative mRNA expression. At the end point, the mean expression of mRNA of PD-L1 (a), PD-L2 (b), and galectin-9 (c) in each group was evaluated using quantitative real-time PCR.

Quantitative real-time PCR showed that the mRNA expression of PD-L1 was significantly lower in the CO_2_-treated and combination groups than in the control group. Similarly, PD-L2 and galectin-9 expression in the CO_2_-treated and combination groups tended to be lower than those of the control and CDDP-treated groups. However, the difference in PD-L2 expression in each group was not statistically significant (Figure 4).

### 3.4. Histological Analysis

**Figure 5:**
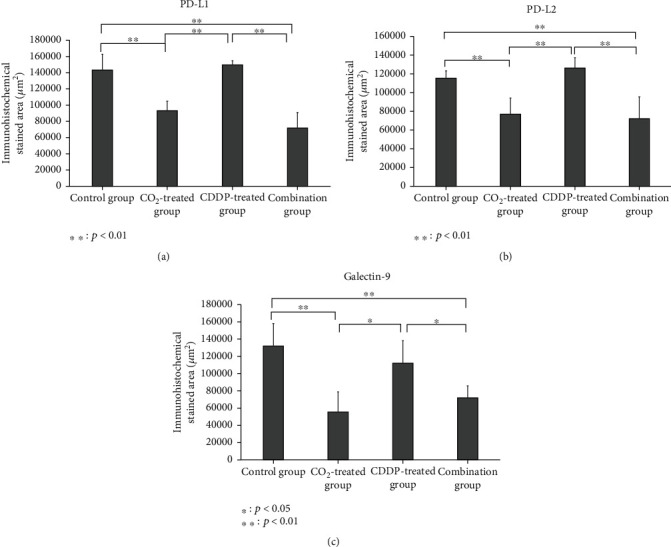
Immunohistochemical staining area. Quantification of mean immunohistochemical staining of PD-L1 (a), PD-L2 (b), and galectin-9 (c). The stained area at each randomly selected point in each group was quantified.

**Figure 6:**
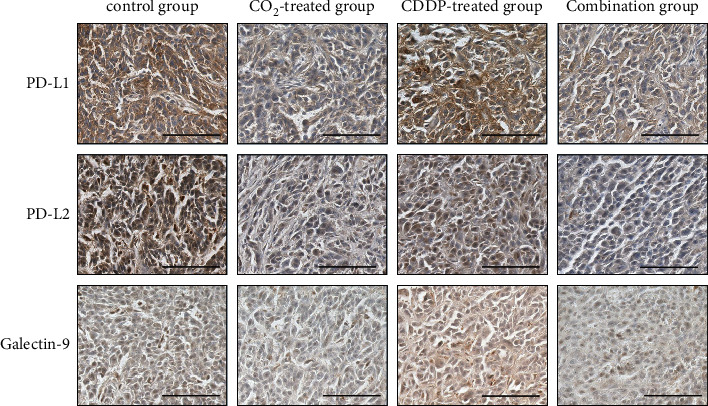
Immunohistochemical staining. Representative histological sections for PD-L1, PD-L2, and galectin-9 in the implanted tumour from each group. Bar = 100 *μ*m.

Consistent with the results of quantitative real-time PCR, immunohistochemical analysis revealed significantly decreased expression levels of PD-L1, PD-L2, and galectin-9 in the CO_2_-treated and combination groups compared to the control and CDDP-treated groups (Figures 5 and 6).

## 4. Discussion

In this study, we showed that transcutaneous CO_2_ application reduced the expression of PD-L1, PD-L2, and galectin-9 in SCC tissues. The combination of cisplatin and CO_2_ application also reduced the expression of these tumour immunosuppressive factors.

To the best of our knowledge, this is the first study on the effects of local CO_2_ administration on chemotherapy and tumour immunosuppression. The negative impact of hypoxia on cancer cells in relation to the efficacy of chemotherapy has been known for several decades [13]. As tumours develop regions of hypoxia, they acclimate through the activation of HIFs, which upregulate the expression of multiple genes associated with angiogenesis, metabolic regulation, pH balance, and cell apoptosis. This results in the promotion of tumour survival [25]. Furthermore, changes in tumour properties by the upregulation of HIFs make solid tumours difficult to treat, leading to resistance to chemotherapy, radiotherapy, and immunotherapy [25].

In recent years, immune checkpoint inhibitors have been shown to affect cancers that are resistant to conventional chemotherapy, and the involvement of tumour immunosuppression in cisplatin-resistant tumours has attracted attention [26]. The PD-1/PD-L1 pathway is one of the representative pathways for tumour immunosuppression in head and neck cancer. Programmed cell death 1 (PD-1) is an immune checkpoint receptor expressed on cytotoxic T cells; there are two ligands, namely, PD-L1 and PD-L2 [27–29]. These are expressed in various cells, including cancer and immune cells, and downregulate T-cell antitumour activity [27–32]. Galectin-9 is a ligand for the immune checkpoint molecule Tim-3, which suppresses antitumour immune surveillance by killing cytotoxic T lymphocytes and impairing natural killer cell activity [33, 34]. There are only few reports on the regulation of galectin-9 expression, and the details of its mechanism remain unclear.

In contrast, there are various reports on the effects of cisplatin and tumour immunosuppressive factors. Many studies have shown that PD-L1 and PD-L2 are upregulated by chemotherapy including cisplatin, but some have shown contrasting results. Ock et al. reported that HNSCC cell lines treated with cisplatin show increased PD-L1 expression [35]. There are also research reports showing increased PD-L1 expression with platinum treatment in other tumours [36, 37]. Sudo et al. reported that cisplatin also increased PD-L2 expression in oral squamous cell carcinoma cell lines [38]. However, no significant change in PD-L1 expression was observed in the chemosensitive cells [13]. In this study, there was no significant difference in the expression of both, PD-L1 and PD-L2, in between the CDDP-treated and control groups. The tumour volume decreased on administration of cisplatin. However, cisplatin did not affect the tumour immunosuppressive factors in this study. In addition, at the end of the intervention, the body weight of the mice in the CDDP-treated and combination groups was significantly reduced compared to that in the control group. Although the dose of CDDP was not considerably higher than those of previous studies [39, 40], it is possible that the observed weight loss was related to the toxicity of cisplatin. These results may be attributed to the type of cell line, the cisplatin treatment regimen, and the time point of evaluation. To evaluate the effects of chemotherapy, it is necessary to examine treatment conditions in further investigations. At least, the findings suggest that CO_2_ administration does not increase the toxicity of cisplatin.

Hypoxia can affect immune evasion in tumours, and several mechanisms have been reported [41]. HIF-1*α*, a transcription factor that promotes the transcription of genes required for adaptation to hypoxia, regulates PD-L1 expression transcriptionally [11, 12]. It is suggested that a similar mechanism is involved in the regulation of PD-L2 expression [42]. It has also been reported that PD-L2 expression is upregulated by GLUT1, which is a target gene of the HIF pathway [42]. These findings suggest that the hypoxic environment in the tumour, chemotherapy resistance, and tumour immunosuppressive effect appear to interact with each other. Therefore, it is expected that improving the hypoxic environment of tumours may reduce the expression of tumour immunosuppressive factors, which may lead to improved tumour chemotherapy resistance. Hence, various methods such as hyperbaric oxygen therapy, hypoxia-activated prodrugs, and oxygen transport agents have been devised to improve hypoxia in the tumour [8]. However, no clinically effective method has been established for improving tumour hypoxia to date [8].

We have previously reported that the transcutaneous application of CO_2_ improves hypoxia in healthy people and various animal models [14, 43, 44]. It has been confirmed that transcutaneous CO_2_ application to the flap on the back of rats improves its blood flow and reduces HIF-1*α* [45]. Transcutaneous CO_2_ application also suppresses the growth of primary human SCC and related lymphogeneous metastasis by making their environment less hypoxic and increasing HIF-1*α* [16, 17]. In this study, it is highly likely that the decrease in PD-L1 and PD-L2 expression in the CO_2_-treated group was caused by the improvement of hypoxia through CO_2_ application. In the combination group, galectin-9 showed similar results as those of PD-L1 and PD-L2. Although the effect of the combined use with cisplatin could not be confirmed at this time, the findings suggest that CO_2_ application is effective in reducing tumour immunosuppressive factors that are generally elevated. Based on the findings of this study, we speculate that the combined administration of CO_2_ may reduce chemoresistance to cisplatin by improving hypoxia.

The strengths of the transcutaneous CO_2_ administration method used in this study lie in the fact that it is an inexpensive and simple method, which can efficiently supply oxygen locally. If transcutaneous CO_2_ administration can improve tumour immunosuppression and treatment resistance, it is expected to have many subsequent effects, such as improvement of patient prognosis, increase in survival rate, decrease in drug dose, and reduction of side effects associated with tumour treatment. However, for clinical application, gaseous CO_2_ cannot be applied to the head and neck region; this is a limitation. We are therefore developing a paste, in which CO_2_ is generated; the produced CO_2_ is efficiently absorbed from the skin without the formation of gaseous CO_2_.

## 5. Conclusions

We confirmed that transcutaneous CO_2_ application to a mouse model, in which an SCC was transplanted, reduced tumour immunosuppressive factors such as PD-L1, PD-L2, and galectin-9. This suggests that transcutaneous CO_2_ application improved the hypoxic environment in the tumour and that combined use with chemotherapy may also improve the tumour immunosuppressive mechanism. Further studies are required to confirm the mechanism and clinical effect of CO_2_ application on immunosuppression and chemotherapy.

## Data Availability

The data used to support the findings of this study are available from the corresponding author upon request.
